# Observations on the Chemical Nature of the Humours of the Eye

**Published:** 1804-02-01

**Authors:** Richard Chenevix


### *Observations on the Chemical Nature of the Humours of the Eye. By* RICHARD CHENEVIX, *Esq. F. R. S. and M. R. I. A.*

THE functions of the eye, so far as they are physical, have been found subject to the common laws of optics. It cannot be expected that chemistry should clear up such obscure points of physiology as all the operations of vision appear to be; but some acquaintance with the intimate nature of the substances which produce the effects cannot fail to be a useful appendage to a knowledge of the mechanical structure of the organ.

The chemical history of the humours of the eye is not of much extent. The aqueous humour had been examined by Bertrandi; who said that its specific gravity was 975, and therefore less than that of distilled water. Fourcroy, in his *Système des Connoissances Chimiques*, tells us that it has a saltish taste; that it evaporates without leaving a residuum; but that it contains some animal matter, with some alkaline phosphate and muriate. These contradictions only prove that we have no accurate knowledge upon the subject.

The vitreous humour is not better known. Wintringham has given its specific gravity (taking water at 10000) as equal to 10024; but I am not acquainted with any experiments to investigate its chemical nature.

We are told by Chrouet, that the crystalline lens affords, by destructive distillation, fœtid oil, carbonate of ammonia, and water, leaving some carbon in the retort. But destructive distillation, although it has given us much knowledge as to animal matter in general, is too vague a method for investigating particular animal substances.

I shall now proceed to mention the experiments I have made upon all the humours. I shall first relate those which were made upon the eyes of sheep (they being the most easily procured), and shall afterwards speak of those of the human body, and other eyes. I think it right to observe, that all these eyes were as fresh as they could be obtained.

### SHEEPS' EYES.

### *Aqueous Humour.*

The aqueous humour is a clear transparent liquid, of the specific gravity of 10090,* at 60° of Fahrenheit. When fresh, it has very little smell or taste.

It causes very little change in the vegetable re-active colours; and this little would not, I believe, be produced immediately after death. I imagine it to be owing to a generation of ammonia, some traces of which I discovered.

When

* All these specific gravities are mean proportionals of several experiments. The eyes of the same species of animal do not differ much in the specific gravity of their humours.

When exposed to the air, at a moderate temperature, it evaporates slowly, and becomes slightly putrid.

When made to boil, a coagulum is formed, but so small as hardly to be perceptible. Evaporated to dryness a residuum remains, weighing not more than 8 per cent. of the original liquor.

Tannin causes a precipitate in the fresh aqueous humour both before and after it has been boiled, and consequently shows the presence of gelatine.

Nitrate of silver causes a precipitate, which is muriate of silver. No metallic salts except those of silver, alter the aqueous humour.

From these and other experiments it appears that the aqueous humour is composed of water, albumen, gelatine, and a muriate, the basis of which I found to be soda.

I have omitted speaking of the action of the acids, of the alkalis, of alcohol, and of other re-agents, upon this humour. It is such as may be expected in a solution of albumen, of gelatine, and of muriate of soda.

### *Crystalline Humour.*

To follow the order of their situation, the next of the humours is the crystalline.

This differs very materially from the others.

Its specific gravity is 11000.

When fresh, it is neither acid nor alkaline. It putrifies very rapidly. It is nearly all soluble in cold water, but is partly coagulated by heat. Tannin gives a very abundant precipitate; but I could not perceive any traces of muriatic acid when I had obtained the crystalline quite free from the other humours. It is composed, therefore, of a smaller proportion of water than the others, but of a much larger proportion of albumen and gelatine.

### *Vitreous Humour.*

I pressed the vitreous humour through a rag, in order to free it from its capsules; and in that state, by all the experiments I could make upon it, I could not perceive any difference between it and the aqueous humour, either in its specific gravity (which I found to be 10090, like that of the other,) or in its chemical nature.

M. Fourcroy mentions a phosphate as contained in these humours; but I could not perceive any precipitation by muriate or nitrate of lime; nor did the alkalis denote the presence of any earth, notwithstanding M. Fourcroy's assertion of that fact.

HUMAN EYE.

I could not procure a sufficient quantity of these, fresh enough to multiply my experiments upon them. However, by the assistance of Mr. Carpue, surgeon to his majesty's forces, I fully convinced myself that the humours of the human eye, chemically considered, did not contain any thing different from the respective humours of the eyes I had examined. The aqueous and vitreous humours contained water, albumen, gelatine, and muriate of soda; and the crystalline humour contained only water, albumen, and gelatine. The specific gravity of the aqueous and vitreous humours I found to be 10053, while that of the crystalline was 10790.

EYES OF OXEN.

I found the eyes of oxen to contain the same substances as the respective humours of other eyes. The specific gravity of the aqueous and vitreous humours is 10088, and that of the crystalline 10765.

What is particularly worthy of notice is, that the difference which appears to exist between the specific gravity of the aqueous or vitreous humour and that of the crystalline, is much greater in the human eye than in that of sheep, and less in the eye of the ox. Hence it would appear that the difference between the density of the aqueous and vitreous humour and that of the crystalline, is in the inverse ratio of the diameter of the eye, taken from the cornea to the optic nerve. Should further experiments show this to be a universal law in nature, it will not be possible to deny that it is in some degree designed for the purpose of promoting distinct vision.

In taking the specific gravity of the aqueous and vitreous humours, no particular precaution is necessary, except that they ought to be as fresh as possible. But the crystalline humour is not of an uniform density throughout; it is therefore essential that attention be given to preserve that humour entire for this operation. I found the weight of a very fresh crystalline of an ox to be 30 grains; and its specific gravity was, as I before stated, 10765. I then pared away all the external part, in every direction, till there remained but 6 grains of the centre; and the specific gravity of these 6 grains I found to be 11940. From this it would seem that the density increases gradually from the circumference to the centre.

It is not surprising that the crystalline humour should be subject to disorders, it being wholly composed of animal matter matter of the most perishable kind. Fourcroy says that it is sometimes found osseous in advanced age. Albumen is coagulated by many methods; and, if we suppose that the same changes can take place in the living eye as in the dead animal matter of the chemists, it will be easy to account for the formation of the cataract; a disorder which cannot be cured but by the removal of the opake lens. If a sufficient number of observations were made respecting the frequency of the cataract in gouty habits, some important conclusions might be drawn as to the influence of phosphoric acid in causing the disorder, by the common effect of acids in coagulating albumen.

